# Extraskeletal Ewing sarcoma of the sciatic nerve

**DOI:** 10.1016/j.radcr.2022.12.006

**Published:** 2023-01-12

**Authors:** Daniel Heller, Gabrielle Wasilewski, Jabra Mustafa, Hamza Chaudhry, Emily Lowery, Dariusz Borys, Emad Allam

**Affiliations:** Loyola University Chicago and Loyola University Medical Center, 2160 S 1st Ave, Maywood, IL 60153, USA

**Keywords:** Ewing sarcoma, Extraskeletal Ewing sarcoma, Extraosseous Ewing sarcoma, Sciatic nerve, EES, extraskeletal Ewing sarcoma, EFT, Ewing family of tumors, PNET, primitive neuroectodermal tumor, LCA, leukocyte common antigen, FISH, fluorescent in situ hybridization, EWSR1, Ewing sarcoma RNA binding protein 1

## Abstract

Extraskeletal Ewing sarcoma (EES) is a rare tumor diagnosed in children or young adults and is even more unusual in individuals over 30 years of age. Due to its rare occurrence and low index of suspicion, this tumor can pose diagnostic and therapeutic challenges. We present a case of a 60-year-old male with EES of the sciatic nerve, an unexpected entity given the patient's age, tumor type, and tumor location. This can mimic a nerve sheath tumor on imaging.

## Background

Ewing sarcoma family of tumors (EFT) is a spectrum of neoplastic disease that includes skeletal Ewing sarcoma, extraskeletal Ewing sarcoma (EES), primitive neuroectodermal tumor (PNET), Askin tumor (Ewing sarcoma of the thoracopulmonary region), and rarely visceral tumors [1,2]. EFT are malignant round cell tumors of mesenchymal origin that overall have a predilection for males under 30 years [3,4]. EES is an uncommon Ewing variant that poses diagnostic and therapeutic challenges due to its rarity and nonspecific radiologic findings. Very few cases have reported EES arising from peripheral nerves [3,[5], [6], [7], [8]].

We present a case of a 60-year-old male with EES originating from the left sciatic nerve, the diagnostic workup, treatment plan, and a review of relevant literature.

## Case presentation

**Fig. 1:**
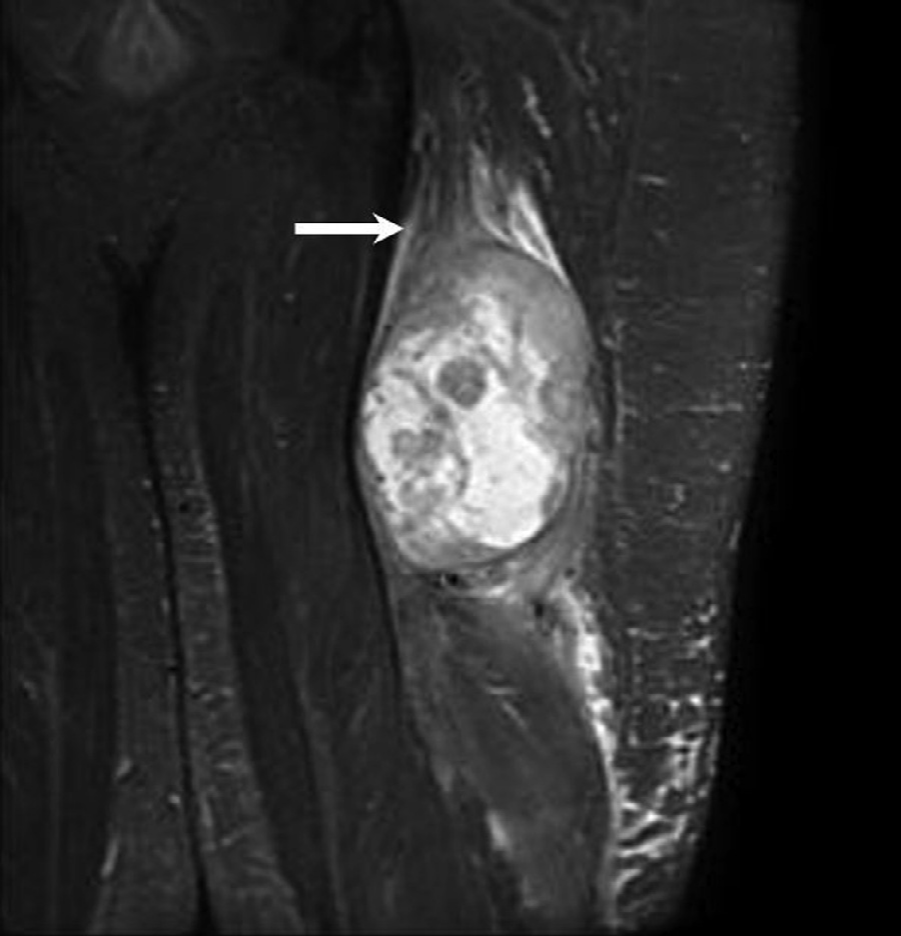
Coronal STIR MRI image shows a heterogeneous ovoid mass in the left thigh with adjacent edema. Proximally, it is continuous with the sciatic nerve (arrow).

**Fig. 2:**
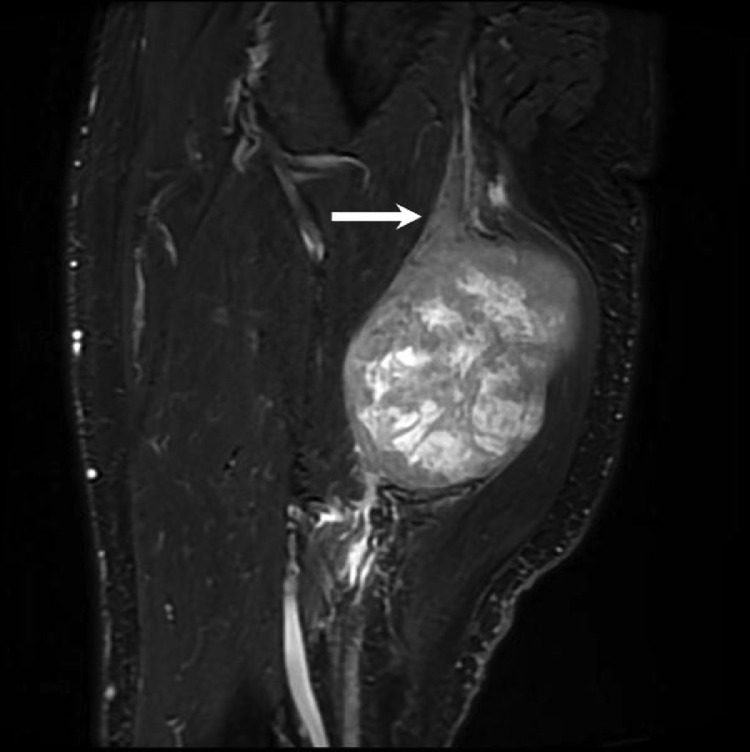
Sagittal T2 fat-saturated MRI image confirms that the mass is continuous with the sciatic nerve proximally (arrow).

**Fig. 3:**
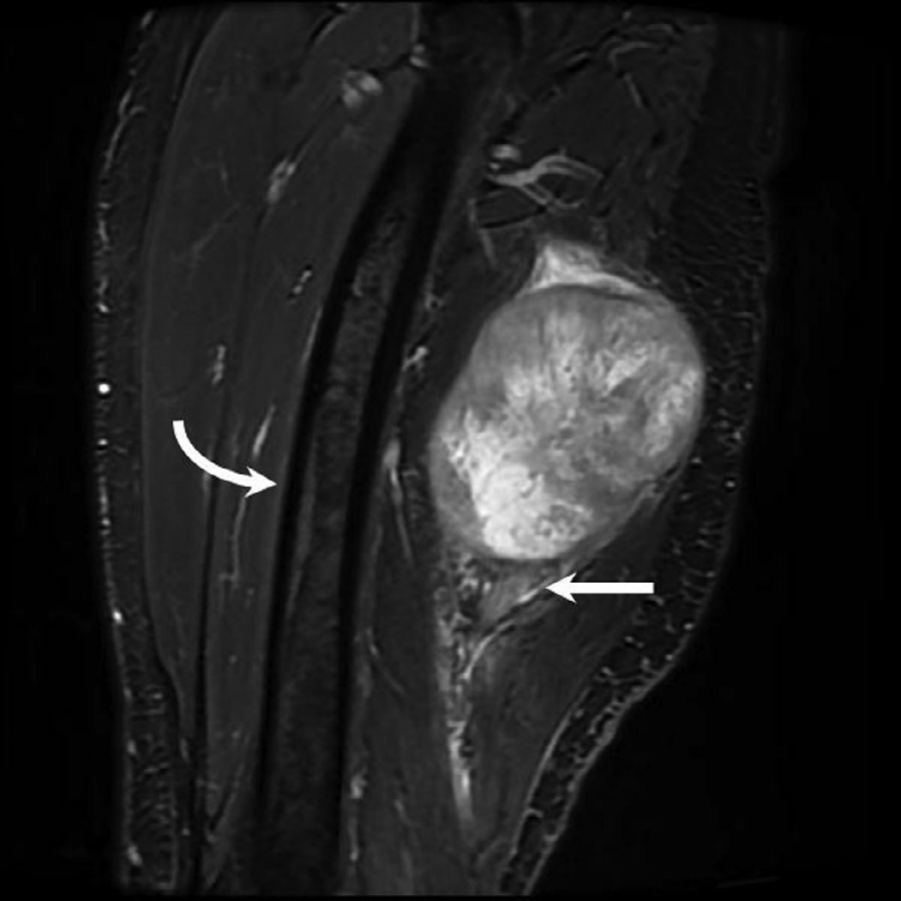
Sagittal T2 fat-saturated MRI image shows the mass is contiguous with the sciatic nerve distally (arrow). There is no osseous involvement of the femur which is anterior to the mass (curved arrow).

**Fig. 4:**
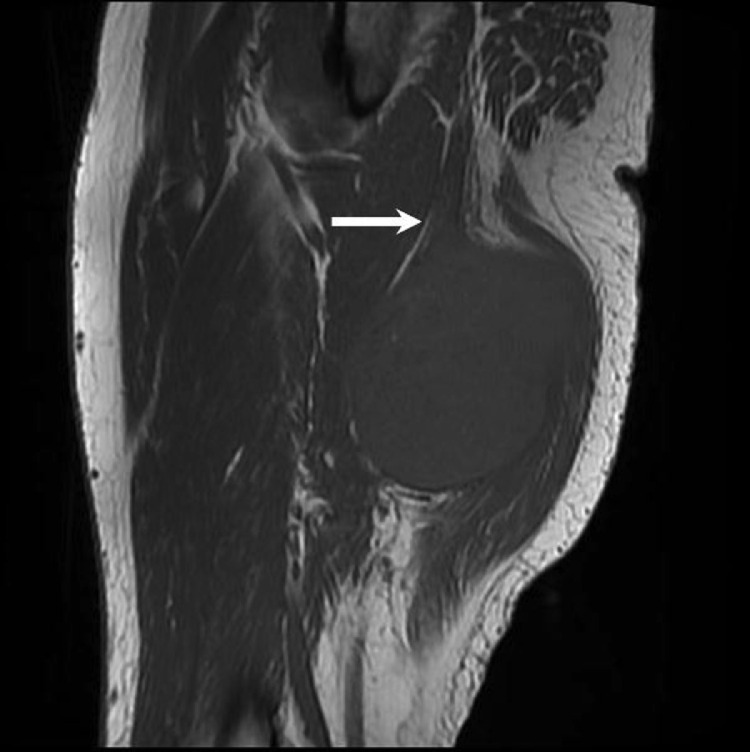
Sagittal T1 MRI image shows isointense T1 signal of the mass relative to skeletal muscle. There is no discernable fat plane between the mass and the sciatic nerve (arrow).

**Fig. 5:**
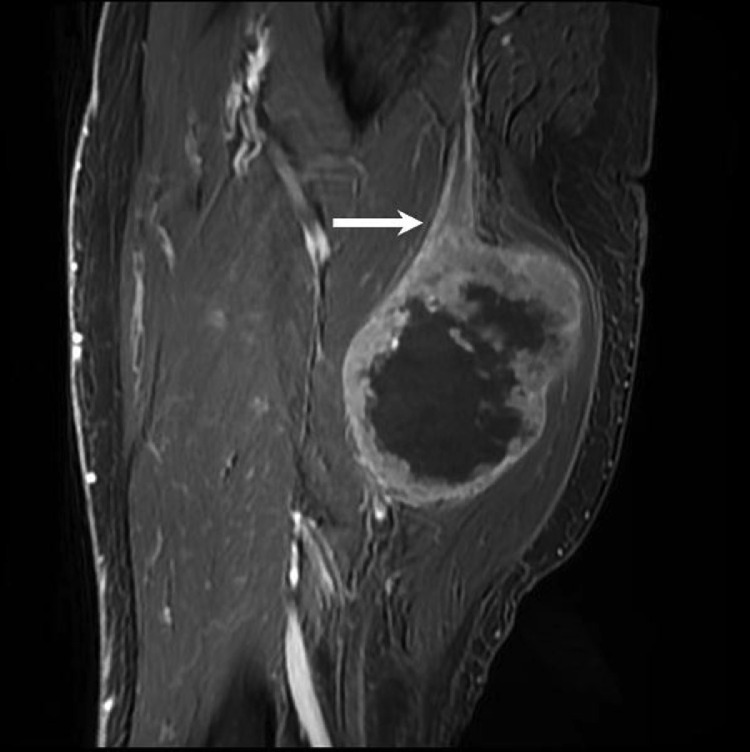
Sagittal T1 fat-saturated MRI image with contrast shows predominantly peripheral enhancement of the mass with extensive central necrosis. There is abnormal enhancement of the sciatic nerve which leads into the mass (arrow).

A 60-year-old male with no significant past medical history presented with a left posterior thigh palpable mass, swelling, and pain. He also reported paresthesias of his left lower extremity. He initially noticed the mass 6 months prior to presentation with interval growth and increasing pain. MRI demonstrated a large necrotic mass in the posterior compartment of the left proximal thigh which was continuous with the sciatic nerve (Figs. 1-5). This measured up to 11.6 cm in the craniocaudal dimension. There was no bone involvement. Duplex ultrasound demonstrated deep venous thrombosis of the left gastrocnemius vein; the other veins of the left lower extremity were patent.

**Fig. 6:**
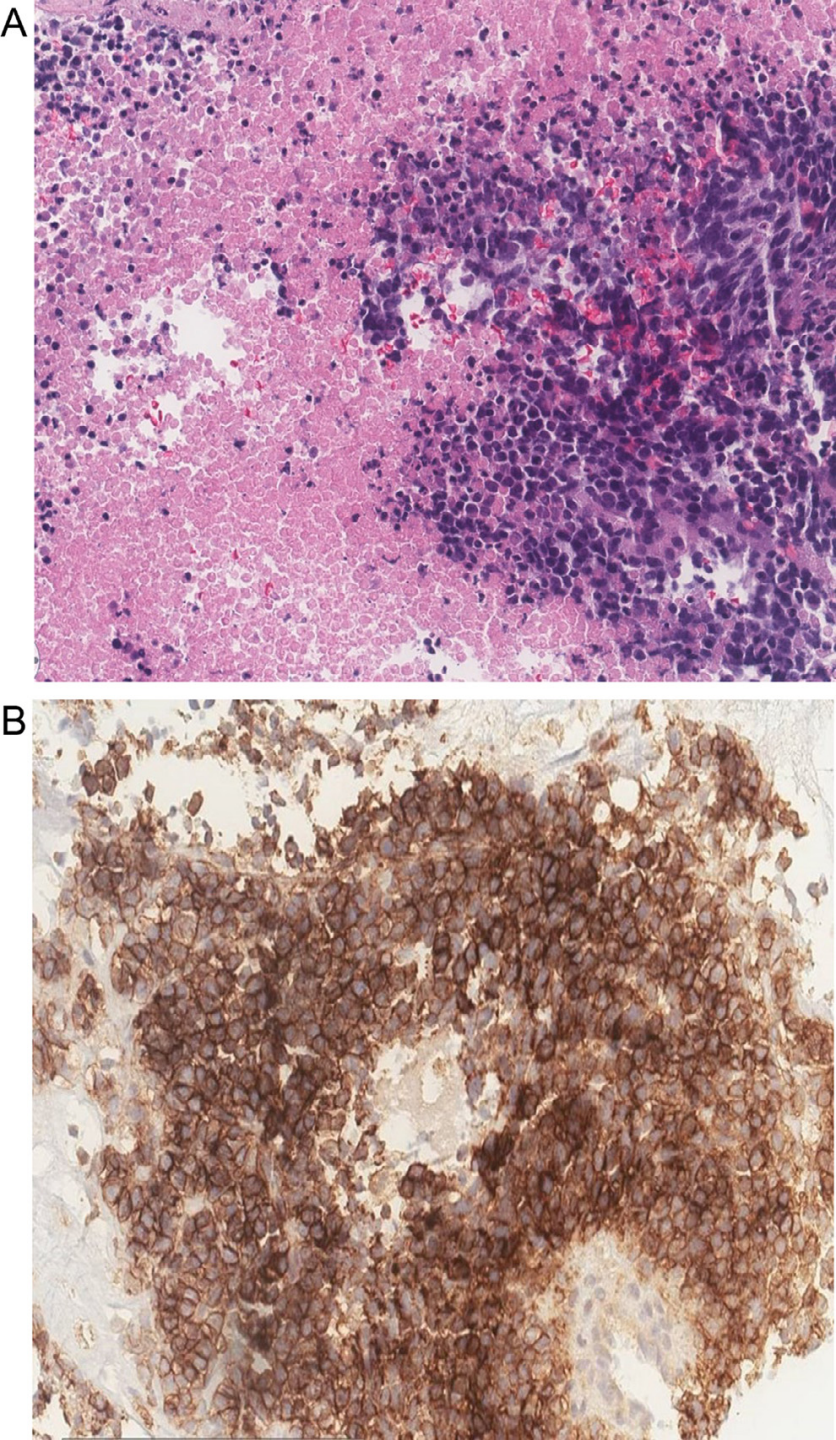
(A) Histopathologic section shows proliferation of poorly differentiated malignant small round cells with increased mitotic figures and necrotic background (H&E stain, magnification 20×). (B) Immunohistochemistry stain positive for CD99 (magnification 20×).

Ultrasound-guided biopsy of the mass was performed. Histopathology showed proliferation of poorly differentiated malignant small round cells with increased mitotic figures and necrotic background (Fig. 6A). Tumor cells were positive for CD99 (Fig. 6B) and negative for cytokeratin AE1/AE3, Desmin, Myogenin, LCA, and S100. Histopathologic findings and immunohistochemistry profile were consistent with Ewing sarcoma. Additional fluorescent in situ hybridization (FISH) analysis was positive for EWSR1 gene rearrangement, which confirmed the diagnosis of Ewing sarcoma.

Apixaban was started for anticoagulation. He received chemotherapy including doxorubicin, cyclophosphamide, vincristine and 6 cycles of ifosfamide and etoposide. Chemotherapy was complicated by syncopal episodes, neutropenic fever, and severe fatigue. The patient declined surgery since surgical resection would involve sacrifice of the sciatic nerve. He underwent radiation therapy of 55.8 Gy in 31 fractions.

There was initially a decrease in size and enhancement of the thigh mass with chemotherapy and radiation. However, there was subsequent increase in size of the index lesion with development of a new lesion immediately next to it in the left thigh, new left inguinal and retroperitoneal lymphadenopathy and pulmonary nodules, consistent with disease progression and metastases. He also developed foot drop and diminished sensation to his left leg below the knee. The patient died approximately 1.5 years after the initial pathologic diagnosis, in large part due to respiratory distress and the extent of pulmonary disease. There were large malignant pleural effusions, with immunohistochemical stains on the pleural fluid showing positivity for CD99 and FLI1.

## Discussion

EFT is a rare class of neoplastic diseases that most commonly affects the bone and soft tissues [9,10]. EFT encompasses Ewing sarcoma, EES, PNET, Askin tumor (Ewing sarcoma of the thoracopulmonary region), and rarely visceral tumors [1]. EFT accounts for the second most common primary bone malignancy in children and adolescents, second to osteosarcoma [10,11]. The peak incidence is during the second decade with an overall predilection for Caucasian males [12], [13], [14], [15], [16]. Roughly 250 patients are diagnosed annually with EFT in the United States [14,17].

Although most Ewing sarcomas arise from bone, up to 30% arise in soft tissue resulting in EES [10]. EES is a rare variant that affects older children and young adults with a worse prognosis [4,18]. These tumors most commonly occur in the upper thigh and gluteal region as well as the upper extremities and paravertebral region [4,14,[18], [19], [20]]. Less than 15 cases have described EES originating from peripheral nerves, with the first reported in 1918 [3,8,21]. EES usually presents as a rapidly growing, painful mass with associated motor and sensory dysfunction if there is nerve involvement [5]. Clinically, EES can mimic other pathologies including schwannoma, lymphoma, metastasis, vascular malformation, hematoma, and abscess [3,6]. Given its rare occurrence, low index of suspicion, and nonspecific clinical manifestations, EES poses a diagnostic challenge.

Imaging serves a critical role in the diagnosis, staging, treatment monitoring, and surveillance of EES. Imaging will often identify an aggressive mass suggestive of a sarcoma or nerve sheath tumor, although the exact diagnosis of EES is challenging since it demonstrates non-specific imaging characteristics. The initial diagnostic work-up of Ewing sarcoma often begins with a radiograph. While radiographs of Ewing sarcoma of the bone often demonstrate destructive lesions in long bones with an accompanying soft tissue mass, the extraosseous variant frequently manifests as a large soft tissue mass near bone without osseous involvement [14]. Ultrasound (US) reveals a heterogeneous mass of predominantly low echogenicity with intratumor flow on Doppler [14,19,22,23]. Anechoic areas representing necrosis or cystic fluid may also be present on US [14,22]. Computed tomography (CT) demonstrates a well-demarcated soft tissue mass that is usually isointense to muscle with occasional areas of necrosis or hemorrhage [14,[23], [24], [25], [26]]. Up to 30% of tumors may also present with calcification [14]. As with other soft tissue masses, MRI is the preferred imaging modality due to its ability to better distinguish soft tissue structures. However, MRI can be non-specific with EES typically demonstrating isointense to low T1 signal, heterogeneous intermediate to high T2 signal, and heterogeneous enhancement [14,23,24]. Although not unique to EES, high-flow vascular channels can be seen in up to 90% of cases. Fluid-fluid levels may also be observed due to hemorrhage [14]. Given the variability of imaging findings, biopsy is required for definitive diagnosis of EES.

Definitive diagnosis of EES is often established by CT- or US-guided core needle biopsy and pathologic analysis. Grossly, EES is a soft, lobulated, gray/yellow/tan tumor with occasional cystic, hemorrhagic, and necrotic areas. Microscopic histological analysis confirms EES via monomorphic proliferation of small, round blue cells with large spherical nuclei with scant cytoplasm [19]. Immunohistochemical markers are positive for CD99 in 90% of cases [21]. Recently, cytogenetic evaluation via FISH paired with immunohistochemistry has been recognized as the gold standard for diagnosis. In 85% of EES, a reciprocal translocation between chromosomes 11 and 22 is present, specifically t (11; 22) (q24; q12) resulting in the *EWSR1-FLI1* gene [7,14,[27], [28], [29]]. About 10% of cases possess a translocation of t (21;22) (q22; q12) to produce the *EWSR1-ERG* gene [4,[30], [31], [32]]. Both of these chimeric genes encode proteins that induce aberrant oncogenic factors [14,19,32].

After establishing the diagnosis of EES, evaluation for metastases helps guide therapeutic approach. The primary prognostic factor of EES is the presence or absence of metastases [11,21,33,34]. At presentation, EES presents with overt metastatic disease in less than 25% of cases [35]. Common sites of metastases include the lungs and bone [14,[36], [37], [38]]. Therefore, imaging studies such as chest CT and FDG-PET are imperative to detect metastatic disease. Localized disease has a 5-year survival rate of 70%, while metastatic or recurrent disease has a 5-year survival of 25% [20,39,40]. Older patient age and male gender are associated with poor prognosis [41].

Therapeutic management of EES requires a multimodal approach. Although the mainstay of treatment for EES has been complete surgical excision of the tumor with negative histologic margins, the neurologic morbidity associated with resection of a peripheral nerve EES makes this undesirable. Moreover, if post-resection nerve grafting is performed, post-operative neuroma may hinder management and surveillance of tumor recurrence [42]. Given the inherent radiosensitivity and chemosensitivity of EES, current literature supports subtotal surgical resection of EES when feasible with adjunct radiotherapy and chemotherapy [8,43,44]. In addition to this multifaceted treatment strategy, imaging surveillance is recommended to limit disease recurrence.

## Conclusion

EES is challenging to diagnose due to its rarity and nonspecific symptomatology. Typically seen in children or young adults, EES would be very unlikely in a 60-year-old patient. Undifferentiated pleomorphic sarcoma, liposarcoma, and leiomyosarcoma are the variations more commonly found in the older adult population and thus the diagnosis of EES is unexpected in older patients. With ambiguous clinical and radiological findings, early histological diagnosis should be sought to avoid diagnostic delay and ensure appropriate treatment. To preserve nerve and limb function and since EES is typically radiosensitive and chemosensitive, radiotherapy and chemotherapy may be advised in addition to/rather than surgical resection.

## Patient consent

Written informed consent for the publication of this case report was obtained from the patient's next of kin.
